# Mismatch repair protein loss in breast cancer: clinicopathological associations in a large British Columbia cohort

**DOI:** 10.1007/s10549-019-05438-y

**Published:** 2019-09-14

**Authors:** Angela S. Cheng, Samuel C. Y. Leung, Dongxia Gao, Samantha Burugu, Meenakshi Anurag, Matthew J. Ellis, Torsten O. Nielsen

**Affiliations:** 1 Genetic Pathology Evaluation Centre and University of British Columbia, Vancouver, BC Canada; 2 Baylor College of Medicine, Houston, TX USA; 3 Anatomical Pathology JPN1401 Vancouver Hospital, 855 West 12th Avenue, Vancouver, BC V5Z 1M9 Canada

**Keywords:** Mismatch repair protein, Breast cancer, Tissue microarray, Tumor-infiltrating lymphocytes

## Abstract

**Purpose:**

Alterations to mismatch repair (MMR) pathways are a known cause of cancer, particularly colorectal and endometrial carcinomas. Recently, checkpoint inhibitors have been approved for use in MMR-deficient cancers of any type (Prasad et al. in JAMA Oncol 4:157–158, 2018). Functional studies in breast cancer have shown associations between MMR loss, resistance to aromatase inhibitors and sensitivity to palbociclib (Haricharan et al. in Cancer Discov 7:1168–1183, 2017). Herein, we investigate the clinical meaning of MMR deficiency in breast cancer by immunohistochemical assessment of MSH2, MSH6, MLH1 and PMS2 on a large series of breast cancers linked to detailed biomarker and long-term outcome data.

**Methods:**

Cases were classified as MMR intact when all four markers expressed nuclear reactivity, but MMR-deficient when at least one of the four biomarkers displayed loss of nuclear staining in the presence of positive internal stromal controls on the tissue microarray core.

**Results:**

Among the 1635 cases with interpretable staining, we identified 31 (1.9%) as MMR-deficient. In our cohort, MMR deficiency was present across all major breast cancer subtypes, and was associated with high-grade, low-progesterone receptor expression and high tumor-infiltrating lymphocyte counts. MMR deficiency is significantly associated with inferior overall (HR 2.29, 95% CI 1.02–5.17, *p *= 0.040) and disease-specific survival (HR 2.71, 95% CI 1.00–7.35, *p *= 0.042) in the 431 estrogen receptor-positive patients who were uniformly treated with tamoxifen as their sole adjuvant systemic therapy.

**Conclusion:**

Overall, this study supports the concept that breast cancer patients with MMR deficiency as assessed by immunohistochemistry may be good candidates for alternative treatment approaches such as immune checkpoint or CDK4 inhibitors.

**Electronic supplementary material:**

The online version of this article (10.1007/s10549-019-05438-y) contains supplementary material, which is available to authorized users.

## Introduction

Mismatch repair is a highly conserved mechanism that maintains replication fidelity and mediates DNA damage signaling [1–4]. Key players in this pathway include EXO1, DNA-binding protein RPA, DNA polymerase, and four major proteins that form heterodimeric complexes: MutS—mutS homolog 2 (MSH2) and mutS homolog 6 (MSH6), MutL—mutL homolog 1 (MLH1) and postmeiotic segregation increased 2 (PMS2) [3, 5]. MutS recognizes and attaches to abnormal DNA whereas MutL enhances recognition and facilitates the formation of a repair complex [5, 6]. The MMR pathway not only corrects base pair mismatches and insertion or deletion loops commonly found in microsatellite regions, it is also involved in cell cycle checkpoints and apoptosis [2–4]. Consequently, deficiencies in MMR pathways promote oncogenesis.

The clinical management of MMR is well-established in colorectal and endometrial cancers. Currently, universal testing in colorectal cancer is recommended by the National Comprehensive Cancer Network and the Evaluation of Genomic Applications in Practice and Prevention Working Group [7] as neither Amsterdam criteria nor Bethesda guidelines [8] can entirely identify all mutation carriers [9]. Likewise, supporting evidence is growing for systematic screening of MMR in endometrial cancers, reflecting the similar rates of Lynch Syndrome in patients presenting with endometrial carcinoma and colorectal carcinoma [11–12].

New treatment strategies have recently become available to MMR-deficient breast cancer patients [13]. In 2017, the United States Food and Drug Administration approved the checkpoint inhibitor pembrolizumab for use in advanced MMR-deficient solid tumors of any tissue type [1]. Furthermore, MMR-deficient breast cancers (specifically those with loss of MutL [14]) have been shown to be resistant to aromatase inhibitors but sensitive to palbociclib (a CDK4/6 inhibitor) [14–16].

The MMR DNA damage repair pathway is likely to hold significant clinical relevance since high mutational load tumors correlate with poor survival [17] and endocrine therapy resistance in ER+ breast cancer patients [18]. However, genomic studies have suggested MMR loss is rare in breast cancer (1–2%) [19], making it difficult to assemble sufficient numbers of cases to power meaningful associative or survival studies. To understand the clinical meaning of MMR deficiency in breast cancer, we assessed a large breast cancer tissue microarray series linked to detailed biomarker and long-term outcome data for immunohistochemically determined loss of MSH2, MSH6, MLH1 or PMS2.

## Methods

### Study cohort

This large tissue microarray series linked to clinical outcomes was built from formalin-fixed paraffin-embedded previously frozen tissues using 0.6 mm cores. Material collection was approved by the Clinical Research Ethics Board of the University of British Columbia (H17-00509), and the characteristics of this cohort have been published [20–22]. Briefly, this cohort comprises 3992 female patients from the province of British Columbia referred to the British Columbia Cancer Agency and diagnosed with primary invasive breast cancer from 1986 to 1992. Patients were treated according to the provincial guidelines in place during the study era [23], and the median follow-up time was 12.5 years. Data for comparative biomarkers on this series have been published: ER [23, 24], PR [23, 25], HER2 [23, 26], CK5/6 [23], EGFR [23], Ki67 [27], PD-1 and PD-L1 [28] (Refer to Supplemental Table A for antibody clones and scoring criteria) and tumor-infiltrating lymphocytes (as assessed on hematoxylin and eosin slides using standardized methods[20].

### Immunohistochemistry

Array sections at 4 μm were mounted on charged glass slides and baked for an hour at 60 °C to prepare for staining on the Ventana Discovery automated stainer (Ventana Medical Systems, Tuscon, AZ). Protocols were adapted from Nordic immunohistochemical Quality Control (NordiQC) [29]. Slides were processed according to manufacturer’s protocol with proprietary reagents. Cell Conditioning 1, heat-induced antigen retrieval, and the discovery anti-HQ HRP detection kit from Ventana were used on all slides; only the dilution and incubation time varied with each biomarker.

Slides were incubated with MSH2 (mouse monoclonal G219-1129; Cell Marque: CMQ-286M14, 1:200 dilution), MSH6 (rabbit monoclonal EP49; Epitomics: AC-0047, 1:50 dilution), MLH1 (mouse monoclonal ES05; Leica Biosystems: NCL-L-MLH1, 1:50 dilution), or PMS2 (rabbit monoclonal EP51; Epitomics: AC-0049, 1:20 dilution). Tonsil tissues, as recommended by NordiQC, were included in each run as an external positive control.

Prior to application to the study cohort, the immunohistochemistry (IHC) protocols optimized for the four MMR biomarkers—MSH2, MSH6, MLH1 and PMS2—were run on independent formalin-fixed paraffin-embedded breast cancer tissue microarrays from a smaller training series to confirm staining interpretability in epithelium and stroma. Furthermore, staining patterns from our optimized protocols were comparable to staining patterns of clinically validated protocols from the Vancouver General Hospital on a colorectal cancer resection control, which was IHC confirmed with MLH1 and PMS2 deletions.

### Scoring

Stained slides were scanned with the Olympus BLISS system. MMR protein expression was scored by a pathologist blinded to the associated outcome data. According to published guidelines, only cores with absent nuclear staining in all carcinoma cells in the concurrent presence of positive stromal cell internal controls on the same tissue microarray core were categorized as deficient [30, 31]. Cores with nuclear staining in carcinoma cells and stromal controls were categorized as intact. Biomarkers were reported following REMARK guidelines [32].

### Data analysis

Statistical analysis was performed with IBM SPSS software (version 25.0). Each biomarker was first dichotomized as intact or deficient. Then, MMR status was assessed. A case was either “MMR intact” when all four biomarkers expressed nuclear positivity, or “MMR-deficient” when nuclear positivity was absent from any of the four biomarkers tested, with respective internal stromal controls. Only cases with interpretable results from all four biomarkers were included in the correlative and survival analyses for MMR deficiency (Refer to Supplemental Figure A for case distribution of the entire cohort and Supplemental Table B for distribution of uninterpretable staining).

Correlations of MMR status with key breast cancer biomarkers were assessed by Fisher’s exact test: ER, PR and HER2 as standard subtype biomarkers; CK5/6 and EGFR for basal-like subtype; Ki67 to examine proliferation for Luminal A vs Luminal B subtype; and immune markers PD-1 and PD-L1. Survival analyses were performed by Kaplan–Meier plot with log-rank test.

## Results

### MMR deficiency is rare in breast cancer

**Fig. 1:**
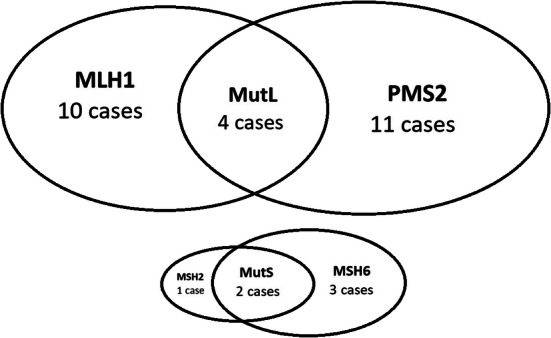
Distribution of MMR-deficient cases

**Fig. 2:**
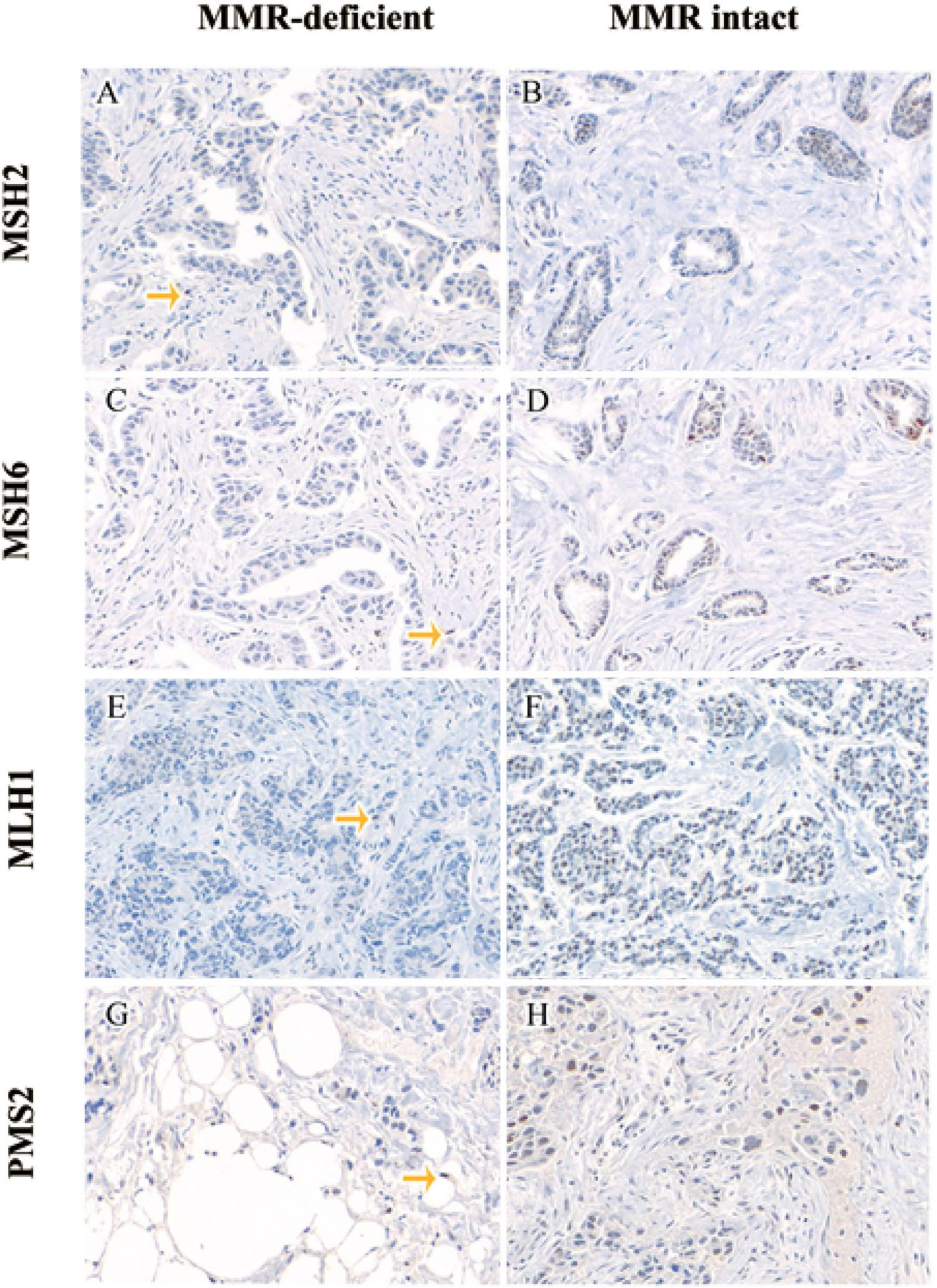
Staining patterns of MMR biomarker: MSH2 loss (**a**), MSH2 intact (**b**), MSH6 loss (**c**), MSH6 intact (**d**), MLH1 loss (**e**), MLH1 intact (**f**), PMS2 loss (**g**), PMS2 intact (**h**). Stromal cell internal positive control in MMR-deficient cases are indicated by gold arrows

Interpretable staining for each individual MMR biomarker (MSH2, MSH6, MLH1 and PMS2) is summarized in Table 1. Of the 1635 cases interpretable for all four MMR biomarkers, we identified 31 cases as MMR-deficient (1.9%). Twenty-five cases had loss of a single MMR biomarker and six cases had paired losses. Four cases had paired MLH1 and PMS2 losses, and two cases had MSH2 and MSH6 losses. Eleven cases had PMS2 loss only, ten cases had MLH1 loss only, three cases had MSH6 loss only and one case had MSH2 loss only (Fig. 1). Figure 2 illustrates examples of MMR-deficient and MMR intact cases.

**Table 1:** Summary of interpretable staining in each biomarker

	Interpretable staining	MMR intact	MMR loss
MSH2	2399 (60.1%)	2363 (98.5%)	36 (1.5%)
MSH6	2488 (62.3%)	2440 (98.1%)	48 (1.9%)
MLH1	1930 (48.3%)	1891 (98.0%)	39 (2.0%)
PMS2	2159 (54.1%)	2115 (98.0%)	44 (2.0%)

Uninterpretable staining includes lack of viable cancer cells, core dropouts from staining or sectioning, insufficient tumour cells and technical fails (apparent loss but without internal positive controls). Refer to Supplemental Table B for detailed distribution

### MMR-deficient breast cancers are likely high grade with low-progesterone receptor expression and high-TIL counts

Among the clinical parameters examined (age, tumor grade, tumor size, lymphovascular invasion, nodal and menstrual status), MMR deficiency was significantly associated with Grade 3 histology (Table 2). We also evaluated associations between MMR-deficient and other important biomarkers previously assessed on this British Columbia cohort (Table 3). Among additional biomarkers with data available for evaluation, MMR deficiency correlated with low-progesterone receptor (PR) expression: 75% (21 cases) of MMR-deficient cases were negative for PR compared to less than half of MMR intact cases. Additionally, cases displaying MMR deficiency had significantly higher TIL counts (median of 5, interquartile range 1–10) compared to MMR intact cases (median of 1, *p *= 0.009 by Mann–Whitney test).

**Table 2:** Association of MMR status with demographic and pathological features

Parameters	MMR loss *n* = 31	MMR intact *n* = 1604	*p* value
Age at diagnosis (years)			
< 50	11	490	N.S.
≥ 50	20	1114	
Tumour grade			
1 and 2	6	636	0.015
3	25	905	
Unknown		63	
Tumour size (cm)			
≤ 2	17	782	N.S.
> 2	14	812	
Unknown		10	
Lymphovascular invasion			
Negative	12	812	N.S.
Positive	17	727	
Unknown	2	65	
Nodal status			
Negative	12	870	N.S.
Positive	19	730	
Unknown		4	
Menstrual status			
Premenopausal	12	504	N.S.
Postmenopausal	19	1067	
Unknown		33	

*N.S.* not significant (*p* value > 0.05)

**Table 3:** Association of MMR status with biomarkers

Parameters	MMR loss *n* = 31	MMR intact *n* = 1604	*p* value
ER			N.S.
Negative	9	478	
Positive	22	1125	
Unknown		1	
PR			0.004
Negative (< 1%)	21	718	
Positive (≥ 1%)	7	825	
Unknown	3	61	
Her2 (erbb2)			N.S.
Negative	25	1325	
Positive	5	248	
Unknown	1	31	
Krt5/6 (CK5/6)			N.S.
Negative	22	1308	
Positive	4	160	
Unknown	5	136	
EGFR			N.S.
Negative	26	1233	
Positive	2	250	
Unknown	3	121	
Ki67			N.S.
Negative (< 14%)	12	754	
Positive (≥ 14%)	15	764	
Unknown	4	86	
Subtypes			
Luminal A	10	616	N.S.
Luminal B	12	494	
HER2E	2	134	
Triple negative/basal	4	275	
Indeterminate	3	85	
PD-1			
Intra-epithelial TIL			N.S.
0	28	1357	
≥ 1%	2	184	
Unknown	1	63	
PD-L1			
Negative (< 1%)	20	1356	0.059
Positive (≥ 1%)	6	165	
Unknown	5	83	
H&E sTILs count (%)			
Median	5	1	0.009^a^
Interquartile range	1–10	1–5	
Unknown	3	97	

*N.S.* not significant (*p* value > 0.05)

^a^Mann–Whitney test

### MMR deficiency is present at similar frequencies across all major subtypes

As basal-like breast cancers are known to have a higher mutational burden than other intrinsic subtypes, and mismatch repair deficiency has been shown to be associated with genomic instability, we analyzed the distribution of MMR-deficient cases by subtype. Results demonstrated no significant differences in MMR deficiency across all the major intrinsic subtypes of breast cancer, as determined by immunohistochemistry (Table 3). Briefly, cases with ER or PR positivity (≥ 1%) and Her2 negativity and low Ki67 (< 14%) were classified as Luminal A; cases with ER+/PR+/HER2−/high Ki67 or ER+/PR+/HER2+ were classified as Luminal B; cases with HER2 positivity with ER and PR negativity ***cases were classified as HER2 enriched; ER−/PR−/HER2− were classified as triple negative or basal if CK5/6+ or EGFR+. The frequency of MMR-deficient cases ranged from 0.5% in basal breast cancers to 2.4% in Luminal B.

### Univariable survival analysis

**Fig. 3:**
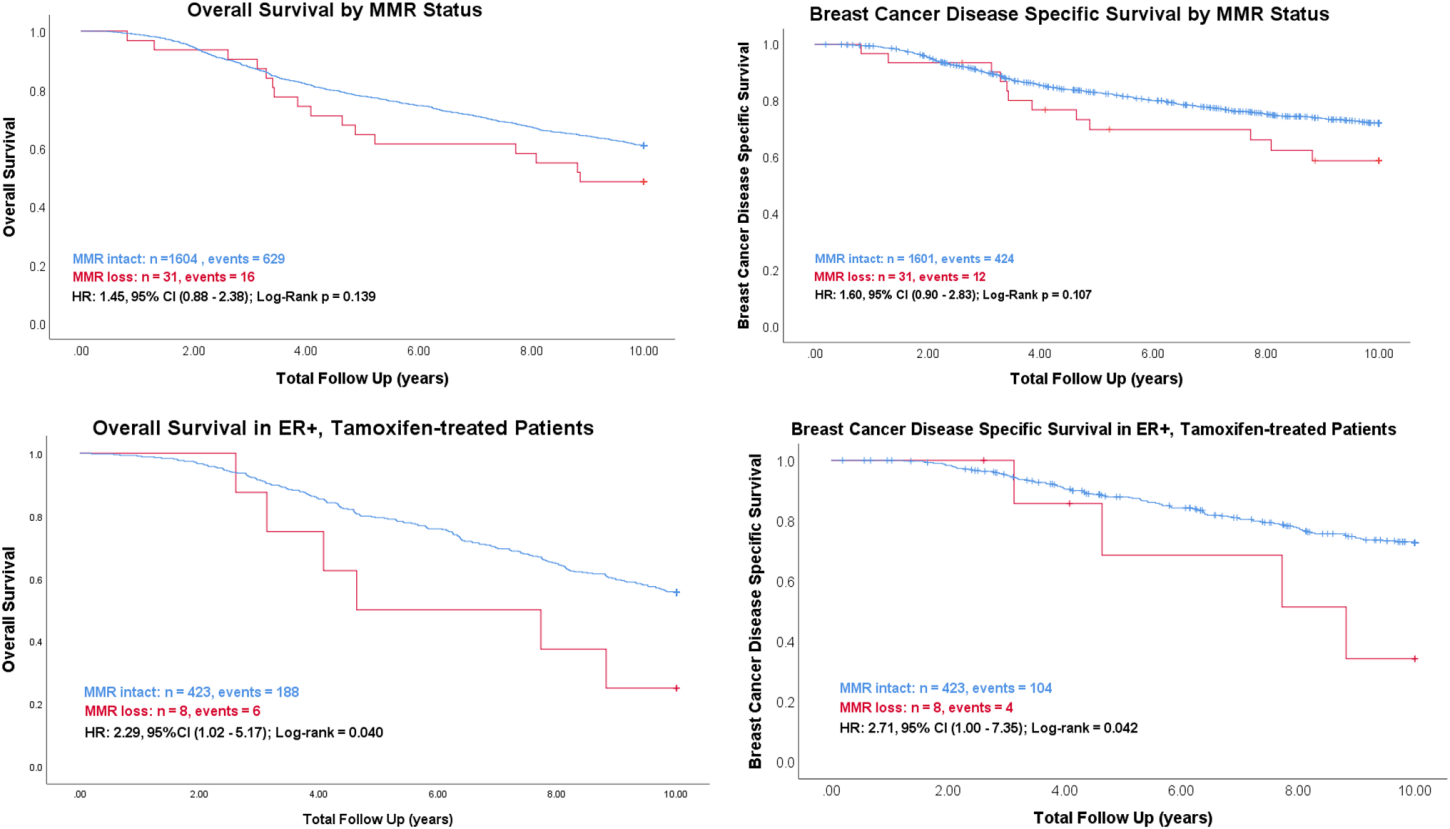
Overall survival (**a**) and breast cancer disease-specific survival (**b**) in the whole cohort. Overall survival (**c**) and breast cancer disease-specific survival (**d**) in ER-positive, tamoxifen-treated patients

As this large breast cancer series had clinical outcome data, we analyzed the prognostic value of MMR deficiency (Fig. 3). Among the whole cohort, overall survival (HR 1.45, *p *= 0.139, *n *= 1635) and breast cancer disease-specific survival (HR 1.60, *p *= 0.107, *n *= 1632) displayed a non-significant decreasing trend with MMR deficiency. The separation of curves was similar for relapse-free survival (HR 1.30, *p *= 0.355, *n *= 1635, Supplemental Figure B). In survival analyses stratified by treatment (Fig. 3), MMR deficiency was associated with significantly shorter survival within the cohort of ER-positive patients treated with tamoxifen as their sole adjuvant therapy (OS: HR 2.29, *p *= 0.040, *n *= 431; DSS: HR 2.71, *p *= 0.042, *n *= 431). Due to the low number of ER-negative cases with MMR deficiency, survival analyses in this population were not conducted.

## Discussion

We present the largest series to date assessing mismatch repair protein deficiency in breast cancer, as determined by immunohistochemistry and linked to survival outcomes. Using our assay, we identified 31 MMR-deficient cases out of 1635 that had data for all four MMR biomarkers (MSH2, MSH6, MLH1 and PMS2). Despite its relative rarity in breast cancer, this population is important as MMR-deficient cancers have been highly responsive to immune therapies such as PD-1 or CDK4/6 checkpoint inhibitors [1, 13, 33–35].

The 1.9% frequency of MMR deficiency in our study corroborates well with the genomic findings reported from the Sanger Centre, who on the basis of mutational signatures derived from whole genome sequencing of 640 cases, identified 11 cases (1.7%) as MMR-deficient [19]. In addition, another study that sequenced MMR genes in 12,019 cancers comprising 32 cancer types reported less than 2% frequency of MMR deficiency in breast cancer [36]. Furthermore, a recent study in 94 HER2-positive luminal B breast cancer patients showed that, although 13.5% of cases had a germline mutation (V384D) in the *MLH1* gene, only 3 cases (3.2%) were MLH1-deficient by IHC [37]. In contrast, a recently published cohort from Italy reported a ten-times higher frequency (17%, 75 out of 444 cases) of homogenous MMR loss by immunohistochemistry [30]; although when further investigated by microsatellite instability assay, all but seven of these were negative (meaning only 1.6% of cases overall were MSI positive). The discrepancy in their reported frequency of MMR-deficient cases in breast cancer by IHC could be due to the inclusion of cases which lack an internal positive control. As this requirement was not mentioned in the reported methods, assessments may be vulnerable to technical false-negative MMR staining, especially when working with clinical cases acquired outside of strict research protocols where variabilities in pre-analytical specimen handling are unavoidable [38]. This discrepancy reiterates the need for IHC assays, which are much less expensive and more widely available than MSI or mutational signature assays, to be standardized and validated with appropriate quality control programs if they are to be put into clinical use [38, 39].

Our results suggest that MMR-deficient cases are associated with poor prognostic factors such as high-grade and high-TIL counts. MMR deficiency is also present across all major breast cancer subtypes by immunohistochemistry in our cohort (1.6% in Luminal A, 2.4% in Luminal B, 1.5% in HER2 enriched, 0.5% in basal), illustrating that MMR deficiency testing is potentially relevant in all major breast cancer subtypes. Poor survival in the ER-positive, tamoxifen-treated cohort suggests MMR status can potentially identify a subpopulation of ER-positive patients that may benefit from treatments beyond endocrine therapy alone.

Our study does have several limitations. Although we examined a large TMA set with extensive published data, each case is represented by a single 0.6 mm core. To avoid overestimating MMR deficiency rates, we only included cases where all four tested MMR proteins had interpretable data not only for carcinoma cells but also for positive stromal cell controls. As cases with apparent MMR biomarker loss without internal positive controls ranged from 230 cases (5.8%) for MSH6 to 523 (13.1%) for MLH1 (Supplemental Table B), the frequency of MMR deficiency we report could be an underestimation, although it does agree quite closely with genomic findings as described above [19, 36]. The biomarker patterns in MMR-deficient cases were not always coherent with expected mismatch repair biology (i.e. some cases showed MLH1 loss with intact PMS2, or MSH2 loss with intact MSH6) [38, 40], a result that may arise from false negatives from tumor heterogeneity that is not adequately assessed using tissue microarrays [41]. Unfortunately, in our series, sequencing data were not available for the great majority of the 1635 cases with MMR IHC results. Although eight positive cases had panel sequencing data available for 83 genes [42], this data was not sufficient to confidently infer MMR genotypic status.

Another limitation of our study is that the determination of MMR loss by IHC is based on its strong correlation with the functionality of MMR rather than direct assessment of DNA mutational patterns. We are aware of the potential misrepresentation, but have found many sources supporting the robustness of IHC compared to genomic methods [19, 30]. Some reports suggest there is a tradeoff of higher specificity achieved through curated genomic methods versus a higher sensitivity using protein expression detected through IHC methods[19]. We opted for IHC because MMR deficiency in breast cancer patients is very rarely hereditary [37, 43]. Additionally, IHC is well-established in colorectal cancer and endometrial cancer, inexpensive and readily available [40, 44].

Our initial cohort comprises 3992 patients. After accounting for inevitable tissue loss from IHC handling, insufficient tumor sampling due to core depletions from sectioning, and exclusion of data from cores without concurrent positive internal stromal controls, we were left with 1635 cases with all four interpretable MMR biomarkers. Since the nature of our TMA resources is one core per case, we lost data on cases that failed to meet the strict MMR-deficient criteria. In the future, one may include additional replicate cores per case to increase the probability of having interpretable stromal controls. Nonetheless, to the best of our knowledge, 1635 cases remains the largest cohort of breast cancer patients examined for mismatch repair protein expression linked to long-term survival data.

Overall, our study reports a low frequency of MMR loss in breast cancer, as determined by IHC, which is present across all major subtypes. Frequencies agree with genomic data, but the IHC approach we used facilitated the examination of large numbers of cases with long-term follow-up, increasing the power to assess the clinical relevance of the relatively rare state of MMR deficiency in breast cancer. There appears to be an association of MMR loss with grade 3, low PR and high-TIL count tumors, as well as with worse survival among ER-positive patients treated with tamoxifen as their sole adjuvant systemic therapy, supporting the concept that patients with such tumors may be good candidates for alternative treatment approaches such as checkpoint or CDK4 inhibitors.

## Electronic supplementary material

Below is the link to the electronic supplementary material.

Supplementary material 1 (DOCX 223 kb)

## Data Availability

The datasets used and/or analyzed during the current study are available from the corresponding author on reasonable request.
