# Supplementary material for: Mismatch repair protein loss in breast cancer: clinicopathological associations in a large British Columbia cohort
Source: Breast Cancer Res Treat. 2019 Sep 14;179(1):3–10. doi: 10.1007/s10549-019-05438-y (PMC6985067; doi:10.1007/s10549-019-05438-y)
Supplement: Supplementary file 1 — Supplementary material 1 (DOCX 223 kb) [file 10549_2019_5438_MOESM1_ESM.docx]

**Supplemental Material**

**Mismatch repair protein loss in breast cancer: clinicopathological associations in a large British Columbia cohort**

Angela S. Cheng BMLSc^1^, Samuel C.Y. Leung MSc^1^, Dongxia Gao MD^1^, Meenakshi Anurag PhD^2^, Samantha Burugu PhD^1^, Matthew J. Ellis MD/PhD^2^ and Torsten O. Nielsen MD/PhD^1^.

^1^Genetic Pathology Evaluation Centre and University of British Columbia, Vancouver, British Columbia, Canada

^2^Baylor College of Medicine, Houston, Texas, US

**Corresponding Author:**

Torsten O. Nielsen, MD/PhD, FRCPC

Anatomical Pathology JPN1401 Vancouver Hospital

855 West 12th Avenue

Vancouver, British Columbia

V5Z 1M9

Canada

604.875.4111 x66768

torsten@mail.ubc.ca

**Supplemental Table A: Summary of antibody clones and scoring criteria.**

| Biomarker | Clone | Dilution | Company | Scoring |
| --- | --- | --- | --- | --- |
| ER | SP1 | 1:250 | Dako | Positive: ≥ 1% tumour nuclei |
| PR | 1E2 | RTU | Ventana | Positive: ≥ 1% tumour nuclei |
| HER2 | SP3 | 1:100 | Dako | Positive: strong complete membrane IHC staining in >10% tumour cells according to HercepTest criteria, with FISH-amplified ratio |
| EGFR | 2-18C9 | RTU | Dako | Positive: any (weak/strong) cytoplasmic and/or membranous invasive carcinoma cell |
| CK5/6 | D5/16B4 | 1:100 | Zymed, Ventana | Positive: any (weak/strong) cytoplasmic and/or membranous invasive carcinoma cell |
| Ki67 | SP6 | 1:200 | Thermo, Ventana | Positive: ≥ 14% tumour nuclei |
| PD-1 | NAT105 | RTU | Roche, Ventana | Positive: ≥ 1 TILs per TMA core |
| PD-L1 | SP142 | 1:100 | Spring Bioscience | Positive: ≥ 1% stained carcinoma cells |
| MSH2 | G219-1129 | 1:200 | Cell Marque, Ventana | Negative: absence of staining in tumour cell with positive internal stromal control |
| MSH6 | EP49 | 1:50 | Epitomics, Ventana | Negative: absence of staining in tumour cell with positive internal stromal control |
| MLH1 | ES05 | 1:50 | Leica, Ventana | Negative: absence of staining in tumour cell with positive internal stromal control |
| PMS2 | EP51 | 1:20 | Epitomics, Ventana | Negative: absence of staining in tumour cell with positive internal stromal control |

RTU: Ready-To-Use

**Supplemental Table B. Distribution of interpretable and uninterpretable staining in each marker.**

**
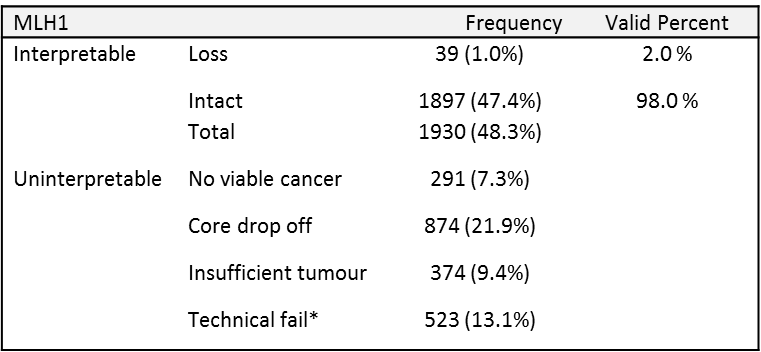
**

**
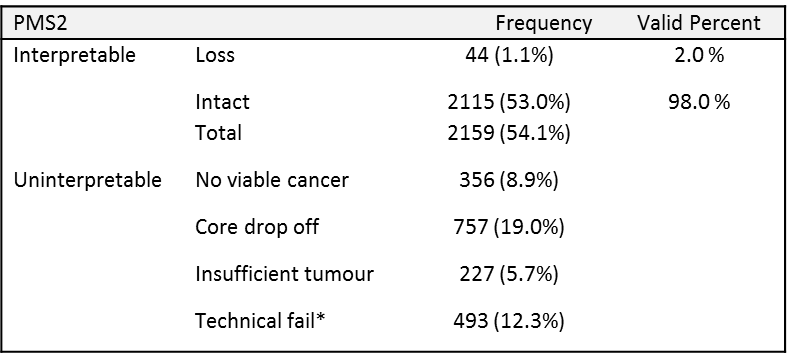
**

**
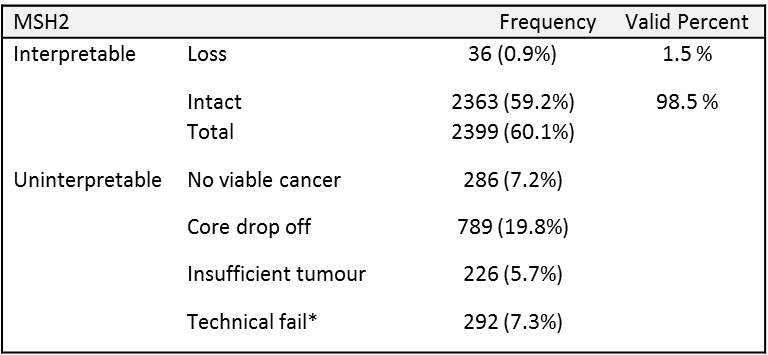
**

**
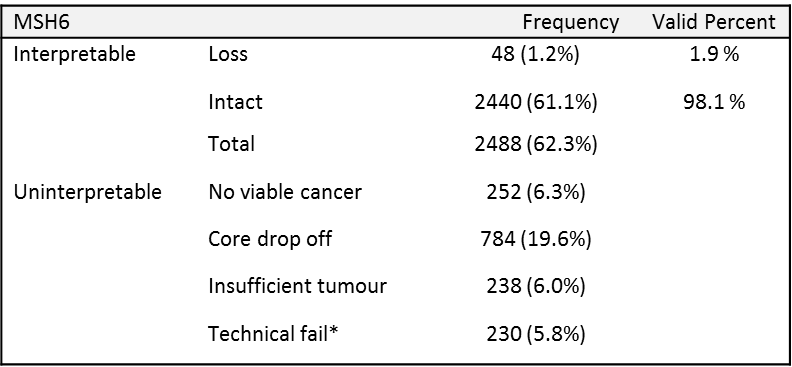
**

*loss without an internal positive control

**Supplemental Figure A.** Case distribution of the entire cohort.


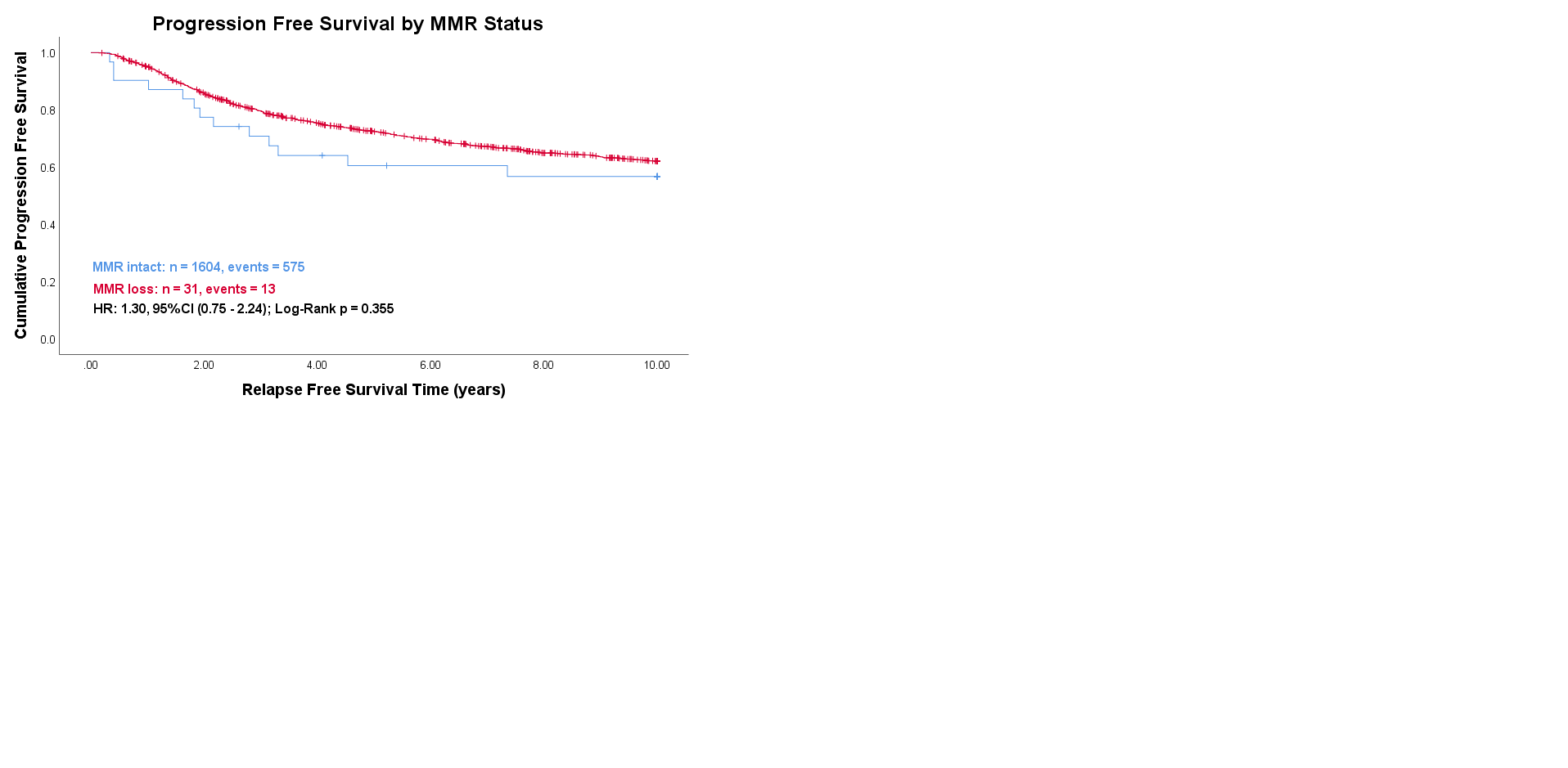


**Relapse-free survival by MMR status**

**Cumulative relapse-free survival**

**Total follow-up (years)**


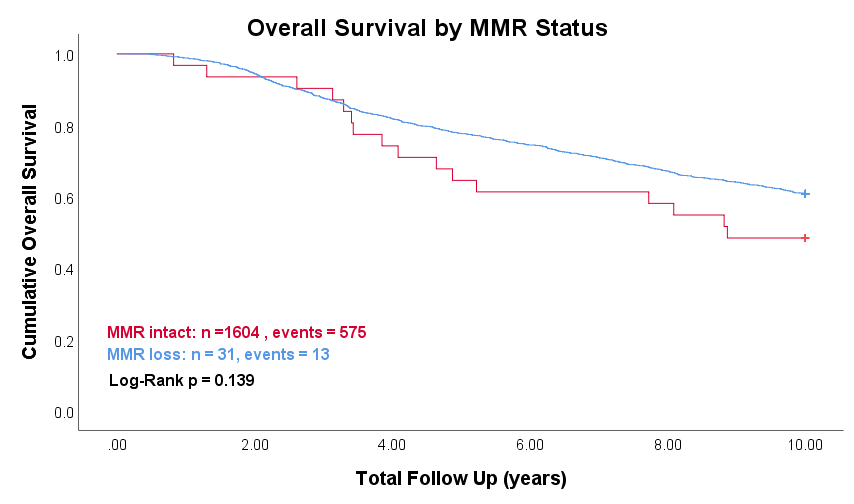


**Supplemental Figure B.** Relapse Free Survival.
